# Multiple uremic tumoral calcinosis in periarticular soft tissues with chronic renal failure: a case report

**DOI:** 10.3389/fendo.2023.1249680

**Published:** 2023-09-08

**Authors:** Lijin He, Meifang Li, Chenlan Lin, Kunlong Yan, Chengmin Yang, Jing Tang

**Affiliations:** ^1^ Department of Radiology, Affiliated Hospital of Putian University, Fujian, China; ^2^ Department of Radiology, West China Hospital of Sichuan University, Chengdu, China

**Keywords:** imaging features, uremic tumoral calcinosis, hemodialysis, surgical excision, recurrence

## Abstract

Uremic tumoral calcinosis (UTC) is an uncommon and severe complication of hemodialysis therapy. The most important pathogenic factor involved in UTC is an increase in calcium-phosphorus products. We report here a patient undergoing hemodialysis for renal failure caused by hypertensive nephropathy who presented multiple UTCs in the right shoulder, left elbow and wrist. After surgical excision, they all recurred, with a similar UTC in the left shoulder. By observing the imaging features of various imaging examinations during the whole period of this case, including X-ray, computed tomography (CT), magnetic resonance imaging (MRI), and single-photon emission computed tomography (SPECT), we highlight the importance of imaging for evaluating the state of UTC regarding treatment options, further deepening our understanding of the imaging manifestations for this disease and their clinical significance.

## Introduction

Tissue calcification in patients on maintenance hemodialysis for chronic renal failure is common. However, uremic tumoral calcinosis (UTC), composed of massive calcium-phosphate deposits, is an uncommon and severe complication, even in dialysis patients, with reported prevalence ranging from 0.5% to 3% (1). Hyperphosphatemia and hyperparathyroidism may largely contribute to periarticular deposition of calcium phosphate, though the mechanism is poorly understood (2). Confirming similar-appearing calcific lesions by imaging alone is challenging, as they share similarities with various differential diagnoses, such as gout, pseudogout, calcific tendinitis, synovial osteochondromatosis, sarcoma, and myositis ossificans (1, 2). Nonetheless, the radiologist plays a critical role in the evaluation of UTC before and after treatment.

We present a case of a 45-year-old Chinese man with renal failure due to hypertensive nephropathy and who had undergone 2 years of hemodialysis, and then developed severe, multifocal periarticular UTC involving the joints of the bilateral shoulders, left elbow and wrist. Our case is unique in that multiple and recurrent UTCs occurred after three surgical removal procedures, confirmed by clinical history, laboratory studies, imaging examination and histopathological analyses. Reviewing the case with full clinical information, especially with respect to imaging features, further deepens understanding of the disease and provides an important basis for diagnosis and treatment.

## Case report

In March 2020, a 45-year-old Chinese man presented to the Orthopedics Department due to the development of progressively enlarging masses. These masses had been present for six months in his right shoulder, four months in his left wrist, and two months in his left elbow. All masses were accompanied by pain and progressively worsened. Importantly, there was no history of trauma associated with these lesions. His past medical history included chronic renal failure (CRF) due to renal hypertension and secondary hyperparathyroidism. Specifically, six years ago, the patient’s blood pressure was noted to be elevated, with a maximum reading of 185/100 mmHg. To manage this condition, a regular prescription of valsartan and levamlodipine benzenesulfonate tablets was initiated. Subsequently, four years ago, an increase in creatinine levels was detected, ultimately reaching approximately 1500 μmol/L, leading to the diagnosis of chronic kidney disease. As a part of the treatment plan, the patient commenced hemodialysis twice a week. After six months of dialysis, urine output ceased, prompting a transition to hemodialysis three times a week. Since initiating hemodialysis, the patient has been receiving calcitriol and calcium acetate to regulate the calcium-phosphorus balance, and cinacalcet was later introduced to address secondary hyperparathyroidism.

**Figure 1:**
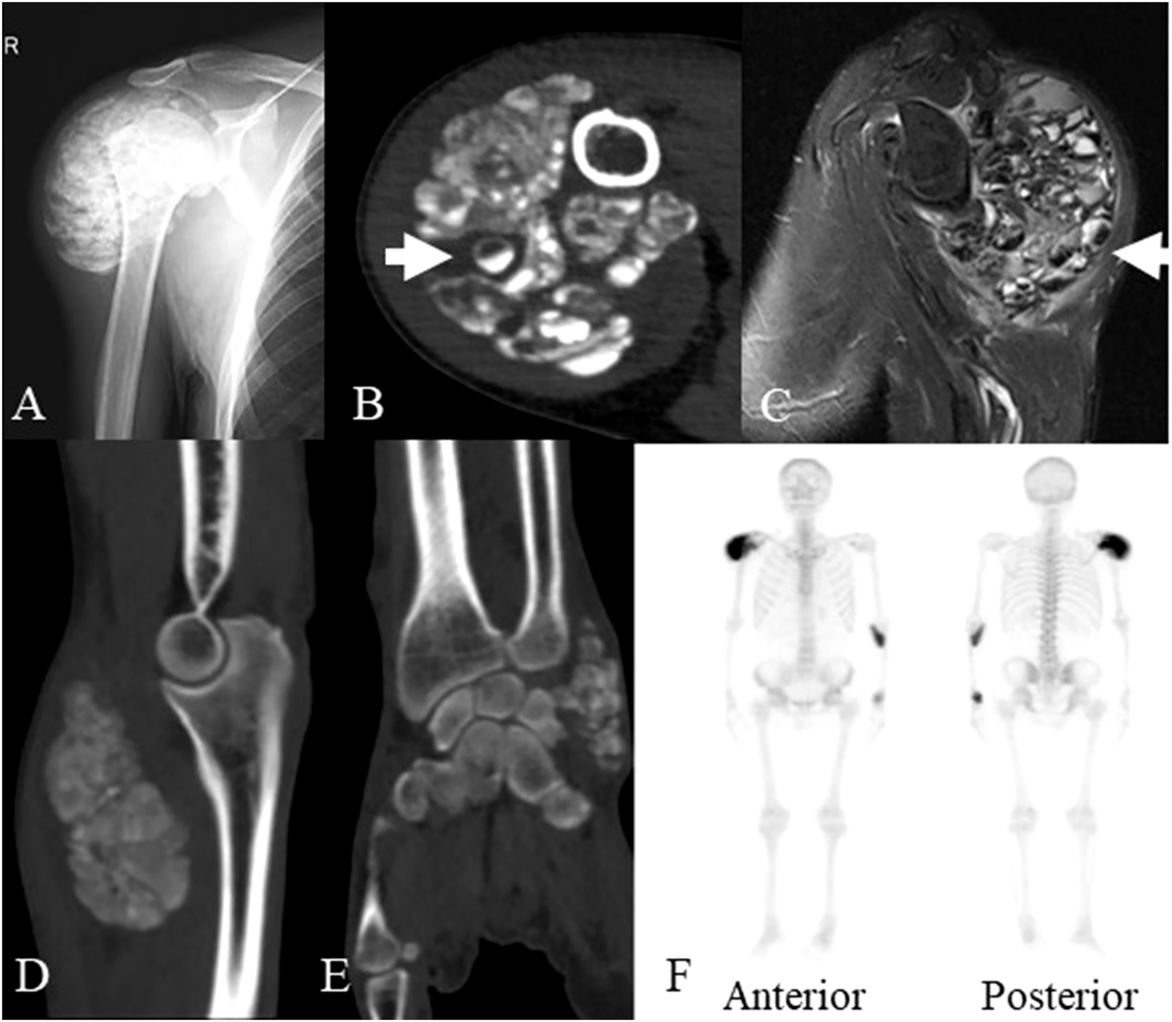
The imaging data of the patient at the age of 45 years old. **(A)** X-ray showed a lobulated calcified mass in the soft tissues around the right shoulder joint. **(B)** Axial CT scan showed a lobulated partially calcified mass around the right shoulder joint, some of which demonstrated cysts with sedimentation sign (arrow). **(C)** MRI showed mixed signal mass in soft tissues around right shoulder joint, some of which also demonstrated cysts with sedimentation sign (arrow). **(D)** Sagittal planes CT scan showed a lobulated calcified mass around the left elbow joint. **(E)** coronal planes CT scan showed a lobulated calcified mass around the left wrist joint. **(F)** SPECT revealed the increased accumulation of radiotracer in the masses of the right shoulder, left elbow and wrist. CT, computed tomography; MRI, magnetic resonance imaging; SPECT, single photon emission computed tomography.

X-ray showed lobulated calcified masses in the soft tissues around the right shoulder, left elbow and left wrist joint with clear boundaries. Computed tomography (CT) imaging revealed lobulated calcified masses in soft tissues around the aforementioned joints with clear boundaries. The density of the masses was not uniform with internal separation, and some of the masses were cystic, with a fluid plane. Magnetic resonance imaging (MRI) showed mixed signal masses in the soft tissues around joints, with high or low mixed signals on T1-weighted image (T1WI) and T2-weighted image (T2WI), also with internal separation and liquid-liquid plane; the lesions invaded the right infraspinatus tendon and the right shoulder joint capsule. Single-photon emission computed tomography (SPECT) indicated hypermetabolic lesions located in the right shoulder, left elbow and wrist (**Figure 1**). The clinical presentation and radiological findings were characteristic of UTC in multiple sites.

Main laboratory analysis included the following: elevated creatinine of 985 µmol/L (normal: 68-108 µmol/L), urea of 29.60 mmol/L (normal: 3.10-8.00 mmol/L), inorganic phosphorus of 2.66 mmol/L (normal: 0.85-1.51 mmol/L), parathyroid hormone of 26.50 pmol/L (normal: 1.60-6.90 pmol/L), thyroid stimulating hormone of 5.08 mU/L (normal: 0.27-4.20 mU/L) and N-terminal fragment of serum osteocalcin of more 300 ng/ml (normal: 14-46 ng/mL). A reduced estimated glomerular filtration rate, 4.91 mL/min/1.73m^2^ (normal: 56-122 mL/min/1.73m^2^), bone-specific alkaline phosphatase of 10.3 µg/L (normal: 11.4-24.6 µg/L) and carboxyl terminal peptide of type I collagen of 2.88 ng/m (normal: 10.30-10.58 ng/m) were also noted. His serum calcium level was normal.

**Figure 2:**
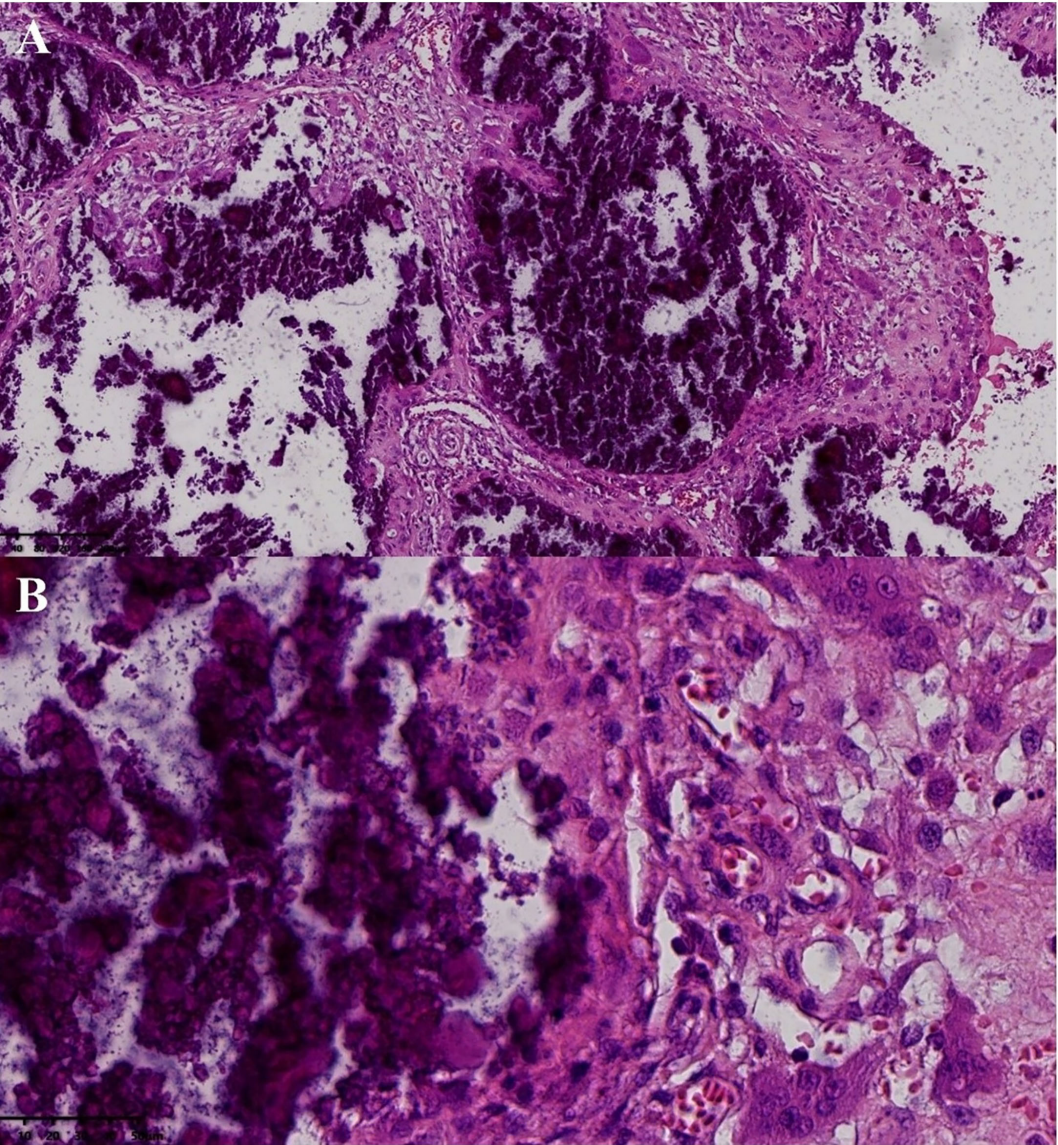
Postoperative pathology of the mass in right shoulder joint. Pathological examination (hematoxvin-eosin stan): an abundance of calcification was observed to accumulate in the masses (**A**, x10), with hyperplastic histiocytes and multinucleated giant cells surrounding the calcification (**B**, x40). Additionally, a substantial amount of fibrous tissue was present between the calcifications **(A)**.

After surgical contraindications were excluded, the first operation was performed for the mass of the right shoulder under general anesthesia. During surgery, a large irregular mass in the deep surface of the deltoid muscle of the right shoulder with a polycystic structure and a large amount of gypsum slurry content with palpable gravel sensation was observed. Postoperative pathology confirmed UTC (active stage) (**Figure 2**). Considering the patient’s poor tolerance of long surgery times, we decided to resect the masses of the left elbow and left wrist joint in the next operation.

At the 4-month follow-up, the patient reported no pain during daily activities in the right shoulder; however, the masses of the left elbow and wrist were gradually growing larger. In July 2020, a second operation was performed on these masses under general anesthesia. During the operation, we found the lesions to be calcium salt deposits and milky viscous fluid, such as gypsum slurry, involving the periarticular fascia and ligaments. Postoperative pathology confirmed UTC. X-ray examination on the second day after the second operation showed incomplete excision. The masses of the right shoulder and left forearm had enlarged after six months, and recurrence was confirmed by CT and MRI. In June 2021, a similar calcified mass appeared in the left shoulder for two months. A third surgery was performed only to resect the mass in the left forearm under general anesthesia. This mass was larger than previous calcifications and infiltrated the flexor space of the proximal left forearm. Postoperative pathology confirmed UTC (active stage).

## Discussion

UTC is secondary and due to CRF, which has been described in hemodialysis patients and may be caused by the inability to normally excrete excess phosphorus from the body through the kidneys as a result of secondary hyperparathyroidism (3). Although the etiopathogenesis of UTC remains poorly understood, it is associated with severe hyperparathyroidism, elevation of serum calcium-phosphorus product or hyperphosphatemia (2). UTC is characterized by slow growing and painless masses of calcium phosphate deposits within periarticular areas. The mass consists of septal, thick connective tissue and cavities filled with a dense milky substance (4), causing functional impairment of the joints or mechanical neural irritation (5).

Histologically, tumoral calcinosis (TC) lesions are dynamic in form and evolution with three clinical stages (2, 6). In the first stage, foamy macrophages aggregate and create a chamber. In the second stage, the lesion is poorly localized and activated and begins to calcify. Then, with the disintegration of calcification, many new small cavities form, and many cavities develop into masses surrounded by fibrous tissues. In the third stage, atrial rupture and fibrosis occur. Radiologic methods can reflect histological characteristics *in vivo* without invasive procedures. In particular, axial CT well delineates a calcific mass and cysts, with the appearance of fluid-fluid levels caused by calcium layering, which is commonly termed the sedimentation sign (7). This is probably due to the granulomatous foreign body reaction or hypervascularity of the lesion (8). The lesion may also appear homogeneous, suggesting reduced metabolic activity and a low likelihood of growth (9). T1WI and T2WI by MRI generally show inhomogeneous high signal intensity and can clearly show sedimentation signs, representing solid, cystic, and fibrous elements with areas of hemorrhage (9). Potential mechanisms underlying the high metabolic lesions detected by SPECT may involve extracellular fluid dilation, heightened local vascular permeability, compromised lymphatic or venous clearance in the affected region, and elevated tissue calcium concentrations (10). These mechanisms may collectively provide valuable information about the active state of the lesion. Combined imaging features could help to distinguish UTC from other diseases that cause calcification of soft tissue around joints, such as gout, synovial osteochondromatosis, acute calcified periarthritis, connective tissue, or degenerative diseases (11).

Treatment of massive periarticular calcinosis largely depends on the underlying cause. Surgical excision of lesions is a well-documented treatment (12), but recurrence due to poor circumscription are common, particularly during active progression. This case highlights an unusual and severe development of multiple UTCs after surgical removal with recurrence and the potential importance of imaging evaluation. There are three aspects of this case that warrant additional discussion.

First, it is well established that in renal failure patients, the occurrence of UTC may be related to impaired calcium-phosphate metabolism (13). In the present case, a series of laboratory tests revealed that serum phosphorus and parathyroid hormone levels were elevated, even though levels of serum calcium were normal. X-ray, CT, MRI and SPECT revealed the status of UTC, which included all clinical stages, such as completely calcified and cysts with sedimentation signs. MRI clearly showed whether the lesion invaded the adjacent tendons and nerves, and SPECT demonstrated the lesions to be metabolic foci. Postoperative pathology confirmed UTC masses at active stage.

Second, three UTC excisions were performed, including complete excisions of the mass in the right shoulder and incomplete excisions in the masses of the left elbow and wrist. Nevertheless, recurrence was observed for all, and a third surgical removal was performed for the recurrent mass of the left forearm. Recurrence of UTC is a frequent complication of incomplete excision, and they typically grow more rapidly than the initial lesion (5). Our case illustrates incomplete excision of lesions, and it is possible that the remaining calcification triggers calcium phosphate deposits in the soft tissue as a localized stimulus. Regardless, with complete excisions of the right shoulder, recurrence was noted. We suggest that active UTC with cysts and sedimentation signs may lead to recurrence, while surgical resection in the quiescent period may reduce the recurrence rate (14). Future work is required to elucidate the basis of the relationship between surgical excision and recurrence.

Third, by reviewing this case, we highlight that imaging contributes to evaluation of the before and after treatment of UTC. Surgical removal of isolated masses has been the preferred treatment (13), however, if an active mass is indicated by imaging or has extensive infiltration into soft tissues and adjacent structures, surgical excision may not be an appropriate treatment due to inevitable recurrence. Other clinical treatments for UTC include dietary phosphorus restriction, calcimimetics, non-Ca-P binders, utilizing low-calcium dialysate solutions, high-flux hemodialysis, parathyroidectomy, and kidney transplantation (15–18). While not yet well standardized, it is important to highlight that in one patient treated with low-calcium dialysis and a non-calcium phosphate binder without increasing the dose of dialysis, the majority of masses disappeared, and the patient became asymptomatic during the follow-up (18). Further research is needed to validate the efficacy of this treatment and its suitable implementation in clinical settings.

## Conclusion

We report a case of multiple and recurrent UTCs after three surgical removals. Although the result in a single case cannot be taken as an indication of effectiveness for UTC, our case suggests the importance of imaging examination before surgical excision. Further investigations are needed to elucidate the recurrence mechanism of UTC.

## Data availability statement

The raw data supporting the conclusions of this article will be made available by the authors, without undue reservation.

## Ethics statement

The studies involving humans were approved by West China Hospital of Sichuan University. The studies were conducted in accordance with the local legislation and institutional requirements. The participants provided their written informed consent to participate in this study. Written informed consent was obtained from the individual(s) for the publication of any potentially identifiable images or data included in this article.

## Author contributions

Conception: JT and LH; Methodological development and statistical analysis: LH, ML, CL, KY and CY; Manuscript draft: LH, ML, CL, KY and CY; All authors contributed to the article and approved the submitted version.
